# Preparation and Performance Study of Microemulsion Acid for Comprehensive Plugging Removal in Carbonate Reservoir

**DOI:** 10.3390/molecules28145606

**Published:** 2023-07-24

**Authors:** Yunjin Wang, Fujian Zhou, Yeping Zou, Zhenhua Wang, Yaocong Wang

**Affiliations:** 1 State Key Laboratory of Petroleum Resources and Prospecting, China University of Petroleum (Beijing), Beijing 102249, China; yunjingwang112@foxmail.com; 2 CNOOC China Limited, Tianjin Branch, Tianjin 300459, China; zyp980415@163.com; 3 Southwest Oil & Gasfield Company Southern Sichuan Gas District, Luzhou 646000, China; wangzhenhua02@petrochina.com.cn; 4 Oil & Gas Technology Research Institute, PetroChina Changqing Oilfield Company, Changqing 710018, China; wyaoc_cq@petrochina.com.cn

**Keywords:** microemulsion acid, plugging removal, acid-rock reaction, wormhole, carbonate reservoir, acidizing

## Abstract

Matrix acidification is one of the most effective stimulations to dissolve scales and remove damage in carbonate reservoirs. However, existing acid systems are difficult to dissolve organic and inorganic scales simultaneously, and complex multi-stage alternative injection often introduces new precipitation and damages the reservoir. Here, based on the retardation ability of emulsified acid and the stable structure of microemulsion, an oil-phase solvent was preferably selected, and the surfactant and cosurfactant were optimized to prepare an acid-in-oil type microemulsion acid capable of dissolving both organic and inorganic scales and high solubilizing for hydrochloric acid. Based on the rotating disc experiment, scale dissolution experiment and acid driving experiment, the acid-rock reaction kinetics, scale dissolution ability and acidizing and plugging removal performance of microemulsion acid in a carbonate reservoir were systematically studied and compared. The results show that Solvesso 150 (aromatic solvent) has the advantages of low toxicity, high flash point and high-scale dissolving ability. At NP−4: OP−10: A (cosurfactant) = 3:3:4, the microemulsion acid system has the strongest ability to solubilize hydrochloric acid and can solve the problem of low H^+^ concentration. The particle size of microemulsion acid is smaller compared to emulsified acid. At 60 °C, the mass transfer coefficient of microemulsion acid is 3.2 × 10^−8^ cm^2^/s, which is one order of magnitude less than that of emulsified acid. Microemulsion acid shows good solubility performance in dissolving organic and inorganic scales, and the comprehensive solubility of mixed scales can reach 98.28%. The stronger scale solubilization ability and lower acid-rock reaction rate enable microemulsion acid to form a thin and straight main wormhole, thus enhancing the acidizing and plugging removal effect. This study can solve the problem of poor hydrochloric acid solubilization ability of microemulsion acid to a certain extent, which provides theoretical and data support for the research and development of microemulsion acid and the efficient plugging removal technology for carbonate reservoirs.

## 1. Introduction

Scaling in the production process of carbonate reservoirs has always been an important issue affecting production and development efficiency [1,2]. Matrix acidizing can fully dissolve the chemical scale generated during drilling and production and can react with carbonate minerals to increase reservoir permeability, thereby enhancing the ability of oil and gas flow [3]. However, the conventional acid system has a single ability to remove scale, and a series of treatment fluids need to be injected alternately to remove various precipitates, but this complex process may produce new precipitates [4].

Microemulsion acid comprises three parts: acid phase, oil phase and surfactant. Most scholars believe that according to the continuous phase and dispersed phase classification, microemulsion can also be similarly divided into the following three categories: acid-in-oil type, acid-in-oil type and double-continuous type [5]. The structure of microemulsion acid and emulsified acid is very similar. Compared with emulsified acid, microemulsion acid is used for smaller particle sizes, so it has stronger stability and better compatibility with additives [6,7]. The surfactant in microemulsion acid is the key to the formation of the microemulsion system. According to the compound surfactants, microemulsion acid can be divided into non-nonionic, cationic-nonionic, and anionic-nonionic microemulsion acid [8]. Alcohols are generally used as cosurfactants in microemulsions, promoting the dissolution of polar substances in the oil phase and enhancing the system’s stability [9]. Hoefner [6] verified that the acid-in-oil microemulsion has a strong retarding performance, but the hydrochloric acid concentration of the microemulsion acid is only 3%. Carvalhoa [10] compared the effects of different surfactants on the three-phase diagram of the system. The final surfactant was Ultrol nonionic surfactant, and the cosurfactants were propanol and butanediol. The concentration of hydrochloric acid in the formed acid-in-oil microemulsion acid increased to 5.12%. The concentration of hydrochloric acid in the existing microemulsion acid system is low. It is an urgent problem to effectively improve the concentration of H^+^ in the microemulsion acid to enhance the ability to remove the plugging and dissolving scale.

There are two main ways to slow down the H^+^ mass transfer rate of hydrochloric acid. One is to increase the viscosity of the acid solution, thereby reducing the diffusion rate of H^+^ [7,11]^.^ The second is establishing an isolation layer on the rock’s surface to isolate the contact between H^+^ and carbonate minerals [12]. However, these two methods require too high injection pressure during treatment, and the scale dissolving ability is poor [13]. The acid-in-oil structure of emulsified acid shows a strong retarding performance. The oil phase isolates the acid phase from the rock surface and reduces the mass transfer rate of H^+^ in the acid-rock reaction, thereby reducing the acid-rock reaction rate [14]. The Almutairi [15] study showed that the smaller the particle size of the emulsified acid, the stronger the retardation ability. The microemulsion acid structure with a similar structure to emulsified acid is more stable. The particle size is at the nanometer level, and the oil of the external phase can also be wrapped in the acid solution [16]. The acid-in-oil microemulsion acid delays the contact between H^+^ and rock, reduces the mass transfer rate of H^+^, and then reduces the acid-rock reaction rate [17]. Different from emulsified acid, microemulsion acid is a thermodynamically stable acid system, which can greatly reduce the acid-rock reaction rate and increase the effective action distance and is more suitable for low-permeability reservoirs with serious pollution [18]. However, at present, the research on acid-in-oil microemulsion acid is still mainly based on retarding performance and drag reduction, and the problem of dissolving mixed scale is almost not considered.

The technology of scale dissolution and plugging removal in oil and water wells are mainly based on an acid system with additives, which is suitable for the demand of plugging removal in oil and water wells caused by inorganic scale, clay particles, serious corrosion and other factors [19,20]. It can effectively remove inorganic scale but cannot remove organic scale. Organic solvent scale remover can effectively remove organic scale but cannot remove inorganic scale [21]. The organic solvent system dissolves the crude oil in the oil sludge by physical extraction according to the mechanism of extraction and plugging removal and the principle of similar phase solubility and disperses and strips the organic heavy components such as colloid asphaltene and paraffin attached to the surface of the oil sludge to achieve the complete separation of the two phases of oil and sludge [22]. Common organic solvents are: xylene, cyclohexane and diesel [23]. The acid solution and the organic plugging remover are incompatible liquids, but the microemulsion can dissolve the two. Therefore, microemulsion with plugging removal has also become mainstream in recent years. Xiao [24] used anionic surfactants to make TC-4 organic plugging remover. The surfactant group can react with the polar groups of organic matter, such as asphalt, to form water-soluble organic salt substances and remove oil-soluble impurities, such as asphalt and paraffin adsorbed on the surface. Lirio [25] developed a single-phase microemulsion for acidizing sandstone reservoirs, which can remove organic and inorganic scales to a certain extent, but did not test the acidizing ability. In general, microemulsion acid with high acidizing ability and the ability to dissolve organic and inorganic scales simultaneously is the trend of plugging and scale removal in the carbonate reservoir.

To solve the problem of removing organic and inorganic mixed scale damage and plugging removal by acidizing technology in carbonate reservoirs, this study optimizes the oil-soluble reagent, optimizes the ratio of surfactant and cosurfactant, and prepares a new microemulsion acid. The microemulsion acid significantly improves its solubilization ability to hydrochloric acid. It has a very low H^+^ mass transfer rate without the need to add other additives that can remove organic and inorganic scales in one step. For the microemulsion acid system, the kinetics of the acid-rock reaction, scale dissolving ability and plugging removal performance in this study.

## 2. Preparation of Microemulsion Acid System

### 2.1. Optimization of Oil Phase Solvent

Most organic plugging in the oil field is composed of asphaltene and paraffin. The experiment uses the high-purity asphalt block to simulate the organic scale. The organic solvent is preferably selected from four solvents: diesel, xylene, cyclohexane, and Solvesso 150 (S150, aromatic solvent).

#### 2.1.1. Experimental Method

**Figure 1:**
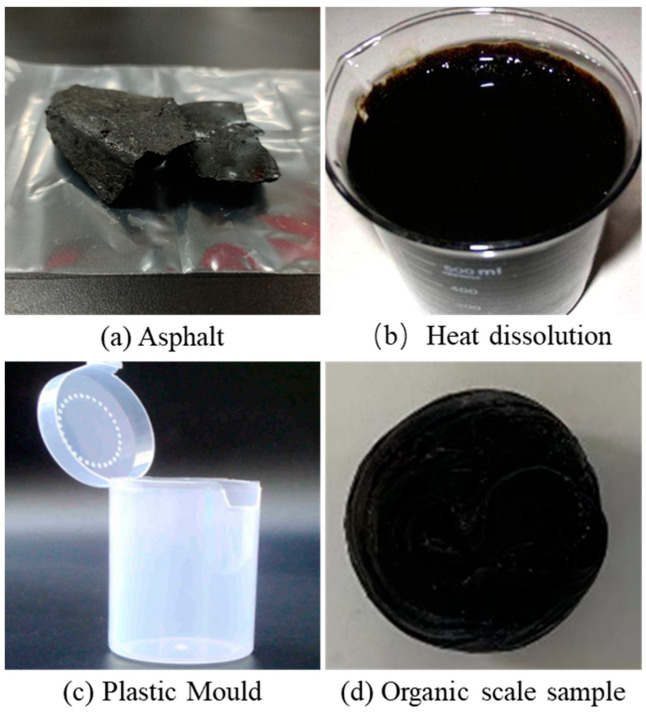
The production process of organic scale sample.

To reduce the influence of surface area on the dissolution rate, spherical asphalt samples with the same surface area were prepared. After the asphalt oil bath was dissolved, the asphalt ball with a mass (*m*_1_) of about 2.5 g was made using a mold, as shown in Figure 1. Put the asphalt balls into the sample tube and add 15 mL of different organic solvents, respectively. Use a constant temperature water bath to heat the sample tube to dissolve the asphalt ball. The dissolved asphalt is filtered out and dried in an oven for 24 h. The remaining dissolved asphalt after filtration was weighed to obtain *m*_2_. The formula for calculating the dissolution rate of asphalt is as follows. To clarify the ability of the oil phase solvents to dissolve asphalt at different temperatures, the water bath temperature in the experiment was changed to obtain the dissolution rate of the oil phase solvents at different temperatures.

(1) $$\mu =\frac{m_{1}-m_{2}}{t}$$

Were *μ* is the dissolution rate of asphalt, *m*_1_ is the initial asphalt ball weight, *m*_2_ is the weight of the remaining asphalt ball, and *t* is the dissolution time of the asphalt.

#### 2.1.2. Experimental Results and Analysis

**Figure 2:**
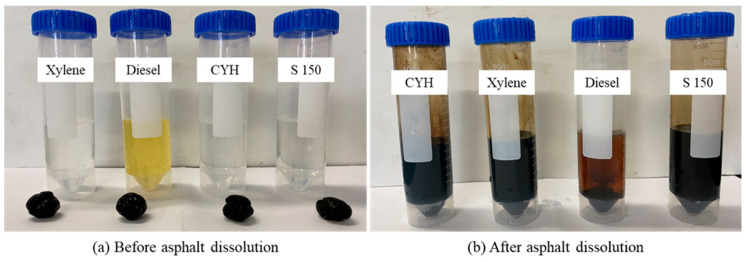
Effect of four organic plugging removers on dissolving organic scale.

**Figure 3:**
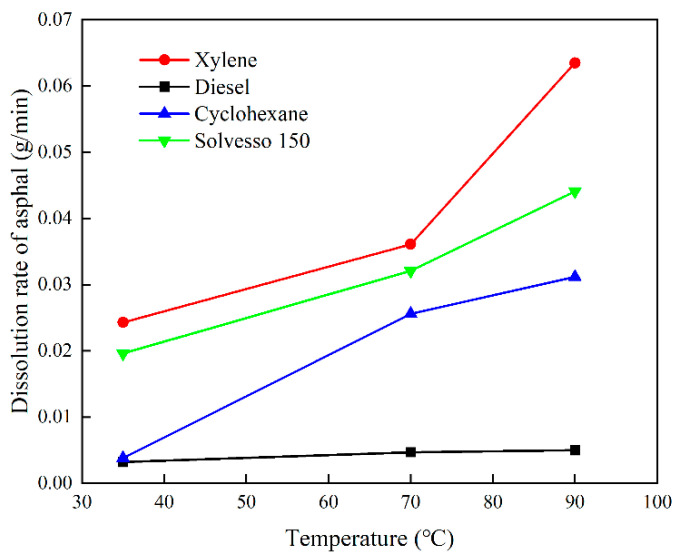
Dissolution ability of 4 organic plugging removers for organic scale.

The sample pictures before and after the experiment are shown in Figure 2. It can be seen from the figure that the colors of the four organic plugging removers become turbid due to the dissolution of organic scales in oil-phase organic solvents. However, it is more obvious that the degree of dissolution of asphalt balls in diesel oil is low, and there are still large asphalt balls after the experiment. The dissolution rate of asphalt in the four organic plugging removers is shown in Figure 3. It can be seen that xylene, cyclohexane (CYH) and S150 have good solubility for asphalt, and the dissolution rate of diesel oil is nearly an order of magnitude smaller than that of them. The increase in temperature has a promoting effect on the dissolution of asphalt. The asphalt dissolution rate of these four organic plugging removers increases with the increase in experimental temperature. The asphalt solubility of diesel oil is weak, only 0.0050 g/min at 90 °C. Xylene, CYH and S 150 have strong asphalt solubility. At 35 °C and 70 °C, the dissolution rates of xylene and S 150 are close.

### 2.2. Preparation of Microemulsion Acid System

Surfactant systems with wide salt tolerance are expected to solve the problem of low H^+^ concentration in acid-in-oil microemulsions [26], and the salinity tolerance of non-nonionic microemulsions is higher than that of cationic-nonionic and anionic-nonionic microemulsions [8], so this work uses a compound non-nonionic surfactant as the surfactant in the microemulsion. Octanol and isopropanol were used as cosurfactant. To have a better ability to dissolve organic scales, S150 was used as the oil phase in this work. Since the amount of acid used in acidizing and plugging removal is usually large, the industry generally uses industrial hydrochloric acid with a concentration of 37% HCl, which is more convenient to purchase. Therefore, the water phase in this work uses a high concentration of 37% HCl to increase the H^+^ concentration of the acid-in-oil microemulsion.

#### 2.2.1. Experimental Materials and Methods

The alcohol used in the experiment is octanol and isopropanol. The surfactants are non-nonionic NP−4 (nonylphenol polyoxyethylene ether) and OP−10 (dodecylphenol polyoxyethylene ether). The oil phase is S150 (aromatic solvent). Acid is 37% hydrochloric acid standard solution. The S + A (surfactant + cosurfactant) phase of the pseudo-ternary phase diagram of the microemulsion acid system consists of nonionic surfactant NP−4 (S_1_), nonionic surfactant OP−10 (S_2_) and cosurfactant (A). Therefore, the ratio of S_1_:S_2_ and the ratio of S:A must be optimized.

At room temperature, the oil phase (O) mass, cosurfactant (A) and the total mass of the two surfactants (S = S_1_ + S_2_) were fixed, the mass ratio of the two surfactants (S_1_:S_2_) was changed, and then added to the beaker on the magnetic stirrer. The hydrochloric acid was added while stirring, and the phase change was observed. Before hydrochloric acid was added, the solution in the beaker was the oil phase. With the gradual addition of hydrochloric acid, the solution changed from the oil phase to the microemulsion phase. When hydrochloric acid increased the solubilizing ability of microemulsion relative to hydrochloric acid, the solution became two-phase. When the phase state was not single-phase, the addition of hydrochloric acid was stopped, and the maximum amount of hydrochloric acid solubilized under the surfactant ratio was recorded. The ratio of S_1_ and S_2_ was optimized according to the mass fraction of hydrochloric acid in the liquid (W_HCl_). After determining the ratio of S_1_ and S_2_, according to the same method, the mass of the oil phase (O) and the ratio of surfactant phase S were fixed, and the ratio of S:A was optimized by changing the total mass of the two surfactants (S) and the mass ratio of the cosurfactant (A).

#### 2.2.2. Ability of Microemulsion to Solubilize Hydrochloric Acid

**Figure 4:**
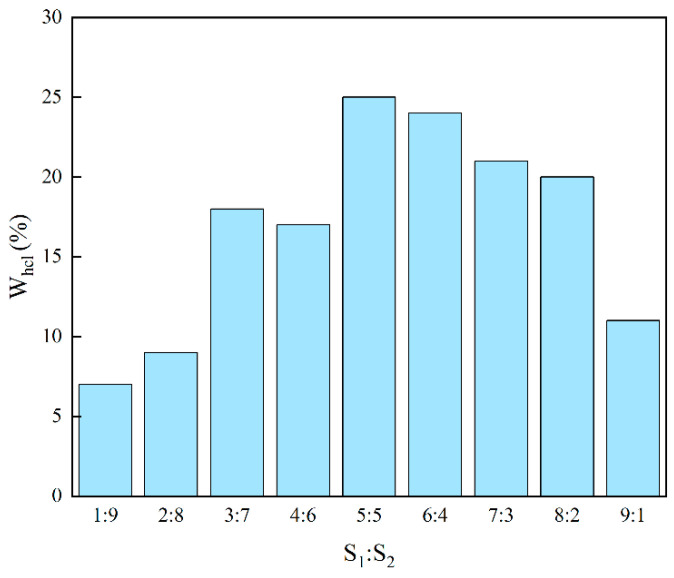
The ability of microemulsion to solubilize hydrochloric acid at different S_1_ and S_2_ ratios.

S_1_ and S_2_ were added to the test tube in different proportions, and hydrochloric acid was added dropwise to observe the process of the system from clarification to turbidity. The amount of hydrochloric acid added at the phase transition point was recorded. The experimental results are shown in Figure 4. The presence of S_1_ and S_2_ greatly enhanced the solubilization ability of microemulsion acid for hydrochloric acid. With the gradual increase of S_1_, the solubilization amount of hydrochloric acid in the microemulsion system increased first and then decreased. When the ratio of S_1_ to S_2_ is 1:1, the solubilization ability of microemulsion acid to hydrochloric acid is the strongest, which is 25%. Therefore, S_1_:S_2_ = 1:1 was selected as the ratio of two nonionic surfactants for preparing microemulsion acid.

**Figure 5:**
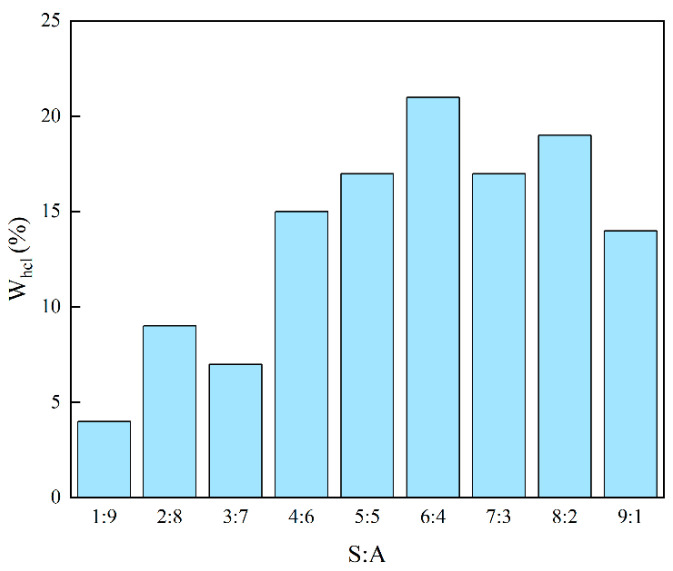
The ability of microemulsion to solubilize hydrochloric acid at different S and A ratios.

After determining the ratio of the two surfactants, it is also necessary to determine the ratio of the compounds S and A. A are generally alcohols, and the amount of A has less effect on the solubilization of the system than S, but it is distributed at the interface between the oil phase and the water phase, which increases the volume of the microemulsion. There may be capacity limitations, so it is necessary to find the optimal ratio of A and S. The experimental results are shown in Figure 5. Compared with the experimental results of non-cosurfactants (Figure 4), cosurfactants can slightly reduce the ability of microemulsion acid to solubilize hydrochloric acid. With the increase of the proportion of S, the solubilization of hydrochloric acid in the microemulsion system also showed a trend of increasing first and then decreasing. When the ratio of S:A is 6:4, the solubilization of microemulsion acid to hydrochloric acid is the largest, which is 21%. Therefore, the ratio of S:A = 6:4 was selected as the ratio of surfactant and cosurfactant for preparing microemulsion acid.

In summary, with Solvesso 150 as the oil phase of the microemulsion and solubilized hydrochloric acid as the standard, the optimal ratio of NP−4 and OP−10 as surfactants and octanol and isopropanol as cosurfactants for the non-nonionic microemulsion acid system was determined as S_1_:S_2_:A = 3:3:4.

#### 2.2.3. Study on Pseudo-Ternary Phase Diagram of Non-Ionic Microemulsion

**Figure 6:**
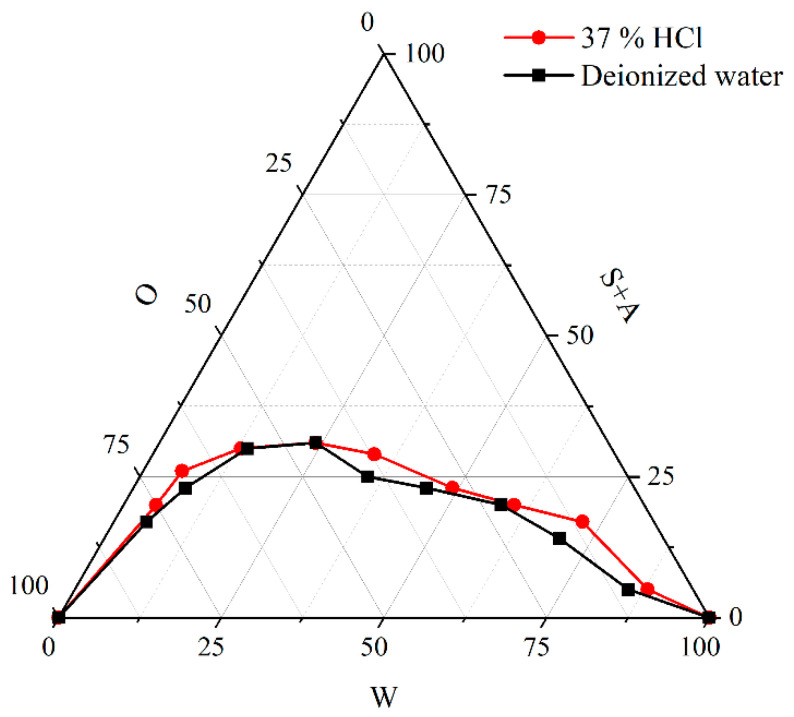
Effect of hydrochloric acid on the pseudo-ternary phase diagram of microemulsion system.

The effect of hydrochloric acid on the single-phase microemulsion region was studied by a pseudo-ternary phase diagram. The impact of deionized water and 37% hydrochloric acid on the system was studied. It can be seen from Figure 6 that the effect of hydrochloric acid concentration on the microemulsion of the system is not obvious. For nonionic surfactants, the existence mode is not ionic state, so the stability is extremely high. Strong electrolytes and acid-base have little effect on the stability of the system. The preparation of the acid system with high-concentration hydrochloric acid will not cause damage to the stability. Therefore, the problem of low H^+^ concentration can be solved by using high concentrations of hydrochloric acid to prepare acid-in-oil microemulsion acid. The formula of microemulsion acid was determined as 36% surfactant phase (10.8% NP−4 + 10.8% OP−10 + 7.2% octanol + 7.2% isopropanol) + 44% oil phase (Solvesso 150) + 20% acid phase (37% hydrochloric acid).

## 3. Study the Reaction Law of Microemulsion Acid

In addition to removing scale, microemulsions can chemically react with the rock minerals of carbonates after entering the reservoir. The comprehensive plugging removal of microemulsion acid to enhance hydrocarbon flow capacity includes plugging removal and reacting with a rock to enlarge the pore structure. The acid-rock reaction rate determines the effective distance of acidizing, which determines the change in permeability and conductivity of the reservoir after acidizing. It ultimately determines the effect of matrix acidizing [27]. A larger acid-rock reaction rate leads to larger acid consumption during acid propagation, which reduces the propagation distance of acid. In this section, the reaction between microemulsion acid and carbonate rock was systematically studied by the rotating disk method, and the mass transfer ability of the dispersed phase H^+^ in the microemulsion acid and the retardation mechanism of the acid system were explored.

### 3.1. Experimental Materials and Methods

The rock samples used in this study are Indiana limestone outcrops, and non-clay and clay mineral X-ray diffraction (XRD) analysis was performed. The results are shown in Table 1. Because of the high calcite content can be regarded as the reaction of HCl and calcium carbonate. Emulsified acid and microemulsion acid with equal H^+^ concentration were prepared to compare the difference in acidizing mechanism between conventional acid system and microemulsion acid. The formulation of the emulsified acid in this work is 30% oil phase (93% S150 + 7% EEA) + 70% acid phase, in which EEA is the surfactant.

**Figure 7:**
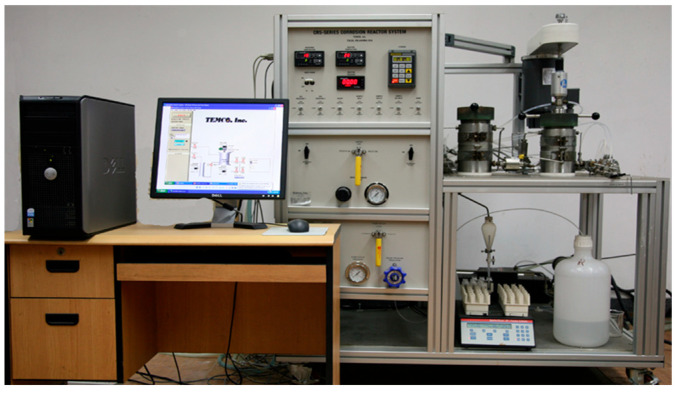
Rotating disc reactor device.

The rotating disk reactor is the most commonly used test equipment, which can be used to test the microemulsion acid system in this study. According to the mass transfer theory of rotating disks, applying a rotating disk reactor is very reliable under clear conditions, and the influence of reaction kinetics and mass transfer motion can be studied completely [28]. The rotating disk reactor used in this experiment mainly comprises a reaction vessel, a liquid storage vessel for storing acid solution, a control panel, and other components. The reaction vessel is 77.30 mm in diameter and 118.10 mm in height, as shown in Figure 7. The reaction vessel in this section can accommodate an acid volume of about 550 mL, using a core sample with a height of 23 ± 0.2 mm. After such a core is installed, the longitudinal distance between the bottom of the reaction vessel and the bottom of the core reaction surface is about 9 ± 0.2 mm.

The experimental steps are as follows: (1) the core was fixed in the reaction vessel using a polytetrafluoroethylene thermoplastic pipe, and the liquid storage vessel containing the acid liquid was preheated. The inert gas N_2_ was added to increase the pressure. The acid in the liquid storage vessel was transferred to the reaction vessel at a pressure of 1 MPa, and the overall pressure was increased to 7.5 MPa. (2) The control panel set the rotational speed to start the experiment. After the experiment, the acid was sampled from the sampling memory immediately, and the samples in the first few seconds were discarded to eliminate the influence of the residual liquid in the sample chamber. (3) 1 mL of acid sample was collected every 10 min 5 times. (4) After the reaction, the core was removed, and the spent acid on the surface was washed with deionized water. The core was placed in an oven at 105 °C for 24 h and weighed.

### 3.2. Reaction Rate of Microemulsion Acid

**Figure 8:**
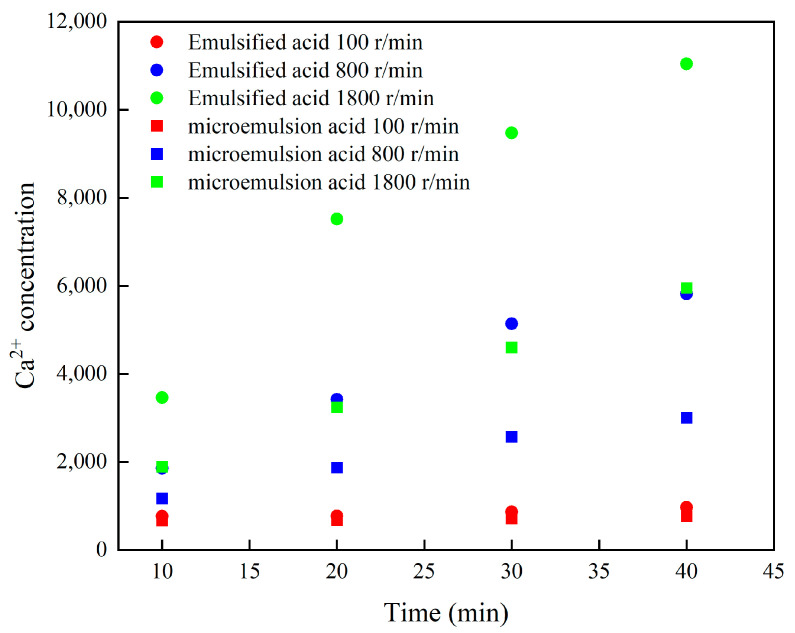
Changes in Ca^2+^ concentration of two acids at different rotational speeds.

Emulsified acid similar to the structure of microemulsion acid was prepared. Three rotational speeds of 100, 800 and 1800 r/min were used to compare the retarding ability of microemulsion acid at 60 °C. The reaction of hydrochloric acid and carbonate rock in the rotating disk is violent, and the core falls off during the reaction process. Therefore, hydrochloric acid is not recorded for comparison. The microemulsion and emulsified acid have a slow reaction rate at 100 rpm, which is lower than the range of the burette, and the fitted linear error may be large. Figure 8 shows the relationship between Ca^2+^ concentration and reaction time in two kinds of acid solution. It can be seen that the mass concentration of Ca^2+^ is linear with time, so the following equation can be used to calculate the mass transfer rate of H^+^ [29].

(2) $$J_{mt}=k_{mt}C_{b}=\left[F(n){\left(\frac{K}{\rho }\right)}^{\frac{-1}{3(1+n)}}{\left(r_{d}\right)}^{\frac{1-n}{3(1+n)}}{\left(\omega \right)}^{\frac{1}{1+n}}D^{\frac{2}{3}}\right]C_{b}=A{\left(\omega \right)}^{\frac{1}{1+n}}$$

Were *K* is the consistency coefficient, *ρ* is the acid density, *r_d_* is the diameter of the rotating disc, *ω* is the rotation rate of the rotating disc, and *n* is a power-law index.

**Figure 9:**
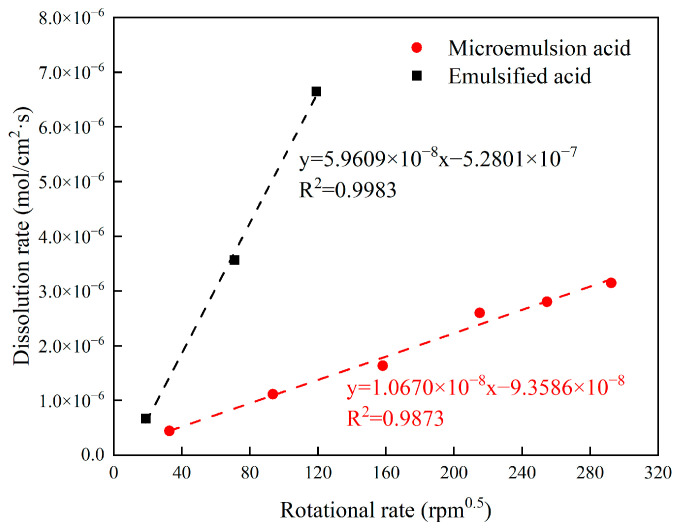
Dissolution rate of two acids at different rotational speeds.

The dissolution rate of carbonate minerals was calculated by Ca^2+^, and the relationship between acid-rock reaction rate and rotational speed was plotted (Figure 9). Overall, the reaction rates of both acids showed an increasing trend with the increase of rotational speed, indicating that under the experimental conditions, both microemulsion acid and emulsified acid were mass transfer-limited types. The reaction of hydrochloric acid with calcium carbonate was significantly greater than that of emulsified acid and microemulsion acid. The dissolution rate of emulsified acid was about two times higher than that of microemulsion acid. The H^+^ of emulsified acid and microemulsion acid need to break through the surfactant layer to enter the oil phase, pass to the rock surface, and react chemically with rock minerals. The particle size of microemulsion acid is smaller, its structure is more stable than emulsified acid, and it is more difficult for H^+^ to break through the surfactant layer, so the dissolution rate of microemulsion acid to carbonate minerals is smaller. When the dissolution process is limited by the mass transfer process, the experimental data can be used to calculate the mass transfer coefficient of the acid solution. Since both the emulsified acid and the microemulsion acid are non-Newtonian fluids, the mass transfer coefficient of the acid solution is calculated by Equation (2).

**Figure 10:**
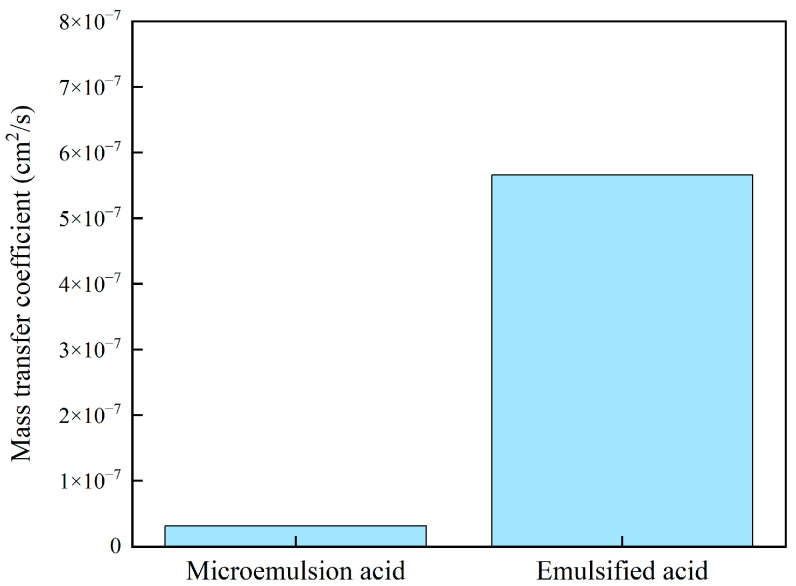
H^+^ mass transfer coefficient of two acid systems.

Figure 10 shows the H^+^ mass transfer coefficient of two acid systems at 60 °C. For the H^+^ mass transfer process, the limitation of the microemulsion structure of the acid-in-oil on the H^+^ mass transfer rate is stronger than that of the emulsified acid. The mass transfer coefficient of microemulsion and emulsified acid are 3.2 × 10^−8^ cm^2^/s and 5.7 × 10^−7^ cm^2^/s at 60 °C, respectively. The mass transfer coefficient of microemulsion acid is more than one order of magnitude smaller than that of emulsified acid. It can be clarified that the mechanism of microemulsion acid reducing the acid-rock reaction rate is similar to that of emulsified acid. The acid liquid is wrapped by the external oil phase to reduce the diffusion rate of H^+^, and the microemulsion acid has a more stable structure than the emulsified acid, further reducing the diffusion rate. Therefore, the retarding performance of microemulsion acid is stronger than that of emulsified acid.

**Figure 11:**
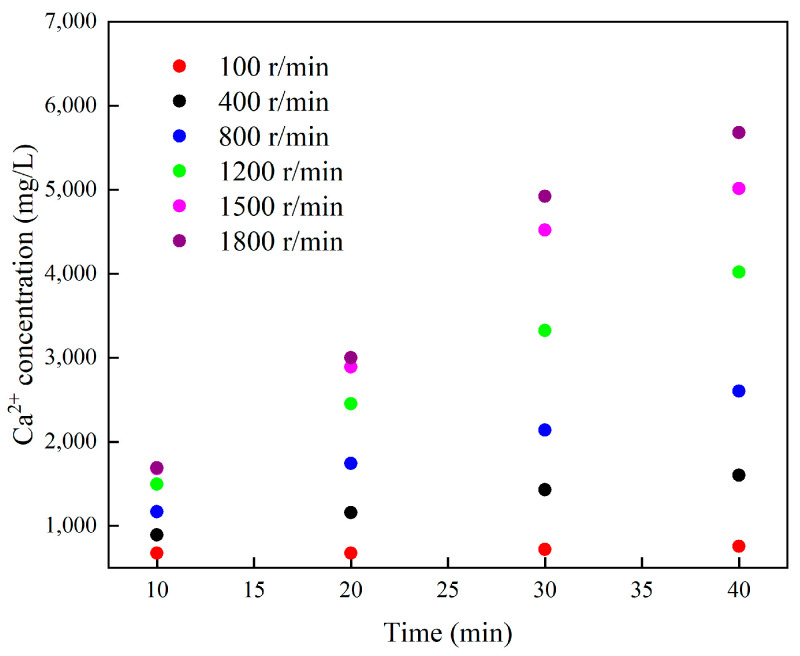
Changes in Ca^2+^ concentration of microemulsion acid at different rotational speeds.

Figure 11 shows the relationship between Ca^2+^ concentration and reaction time during the reaction of microemulsion acid at different rotational speeds. At all rotational speeds, the Ca^2+^ concentration increased with the increase of time, and the Ca^2+^ concentration was linear with time. The most important thing is that the concentration of Ca^2+^ increases significantly with the increase in rotational speed, which is consistent with the results of Taylor‘s study [30]. This shows that increasing the mass transfer rate can effectively improve the reaction rate of microemulsion acid, and even at a high rotational speed, the mass transfer process is still the main controlling factor limiting the acid-rock reaction.

## 4. Study on Plugging Removal Performance of Microemulsion Acid

### 4.1. Dissolving Scale Ability of Microemulsion Acid

The pollutants in oil and water wells can be divided into organic deposition scale and inorganic deposition scale. Organic sediments are mainly composed of paraffin and asphaltene in crude oil. Inorganic sediments are mainly due to the corrosion of pipelines and equipment, forming iron scales such as iron hydroxide and ferrous hydroxide precipitation. In addition, when the injected water is incompatible with the formation water, inorganic scales will be produced [31].

The inorganic scale sample used in the experiment is a mixture of Indiana core and iron hydroxide powder. The organic scale sample used in the experiment is high-purity asphalt. The experimental solution was 20 g of HCl, S150, emulsified and microemulsion acid with the same H^+^ concentration. The experimental conditions were 90 °C, and the reaction time was 4 h. After the reaction, the reaction was filtered and dried at a low temperature for 24 h to weigh and measure the mass after the reaction. The reaction amount and dissolution rate were calculated by weighing before and after.

The experimental results of inorganic scale dissolution are shown in Table 2, HCl has the best dissolution performance for inorganic scale, and the dissolution rate reaches 94.44%. S150 has almost no ability to dissolve inorganic scales. The dissolution ability of microemulsion acid and emulsified acid to inorganic scale is very close to that of HCl, and the dissolution ability of microemulsion acid to inorganic scale is slightly higher than that of emulsified acid.

It can be seen from Table 3 that according to the principle of similar dissolution, HCl has the smallest amount of dissolution of organic matter, and the ability of emulsified acid to dissolve organic matter is mainly reflected by the external phase of organic solvents. Compared with Solvesso 150 solvent, the dissolution quality of organic scale by microemulsion acid is very close. However, only 44% of the microemulsion acid is S150. That is to say, for the dissolution of asphalt per unit oil, the amount of microemulsion acid is more than twice that of Solvesso 150 solvent. This is due to the synergistic effect of surfactant and oil in the microemulsion acid system, which strengthens the maximum dissolution ability of oil relative to the organic scale. Therefore, preparing organic plugging remover into a microemulsion system is helpful to the performance of itself dissolving organic scale.

According to the method of making an organic scale, the organic and inorganic scales are mixed to make a mixed scale. The experimental solution was HCl, S150, emulsified and microemulsion acid with equal H^+^ concentrations. The experimental conditions were a 90 °C water bath reaction for 4 h. After filtration and drying, the mass after the reaction was weighed to obtain the dissolution rate of the mixed scale.

It can be seen from Table 4 that HCl has the lowest dissolution amount of mixed scale. This is because HCl hardly reacts with asphalt. The calcium carbonate and iron hydroxide in the mixed scale sample are almost completely wrapped by asphalt, isolating HCl from its reaction, making it difficult to dissolve. S150 only dissolves asphaltene, and the inorganic scale particles which are wrapped are dispersed in the liquid and cannot be dissolved. The comprehensive dissolution rate is only 49.69%. Although the emulsified acid has a certain degree of dissolution ability for organic and inorganic scales, due to the proportion of S150 in the emulsified acid being about 30%, the particle size of the emulsified acid is large, and the structure is relatively unstable, it is easy to break the emulsification after the acid-rock reaction. The final result is that the dissolution rate of emulsified acid for mixed scale is only 16.16%. Microemulsion acid for inorganic and organic scales can be effectively dissolved, and the particle size is small. The structure is stable, so the dissolution ability for the mixed scale is the highest, and its dissolution rate can reach 98.28%.

**Figure 12:**
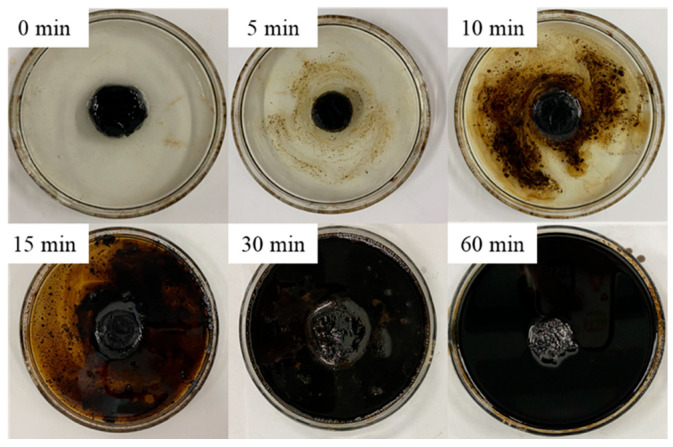
Dissolution process of mixed scale.

**Figure 13:**
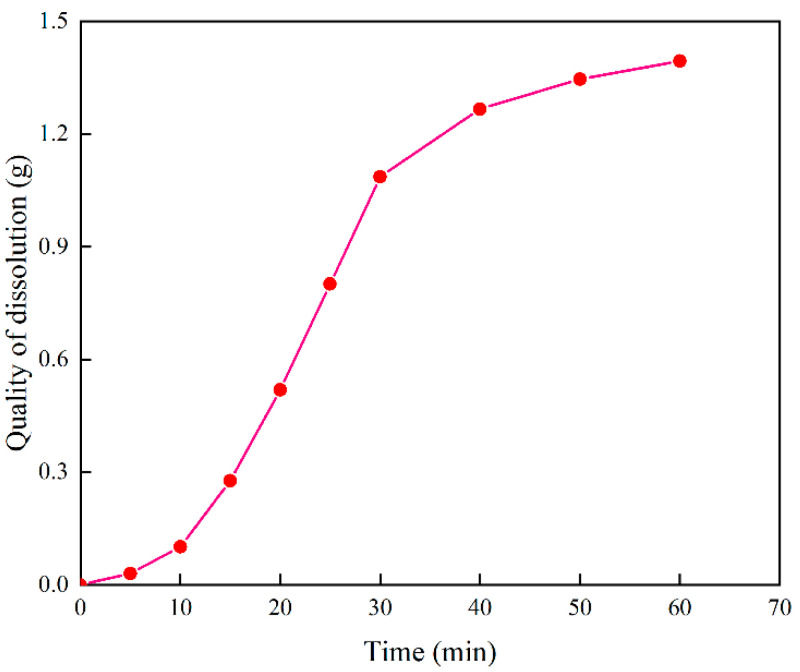
The variation curve of the dissolved amount of mixed scale.

To explore the dissolution mechanism of microemulsion acid for mixed scale, Figure 12 shows the dissolution process of microemulsion acid at room temperature. It can be seen that the dissolution process of microemulsion acid is to dissolve asphalt in the early stage. From the 15th minute, after the surface-wrapped asphalt is dissolved, many bubbles occur with the acid-rock reaction. From Figure 13, it can be seen that the dissolution amount of mixed scale increases steadily from 10 min to 30 min. Initially, the dissolution rate is slow due to the dissolution of only organic scale. When the inorganic scale is gradually exposed and participates in the reaction, the reaction rate increases significantly. With the continuous consumption of reactants, the reaction rate eventually becomes smaller.

### 4.2. Comprehensive Plugging Removal Performance of Microemulsion

The research on the acid-rock reaction mechanism of carbonate rocks has been relatively mature. Acid-etched wormholes can greatly improve the flow conditions of low-permeability reservoirs and improve the effect of acidizing stimulation. The development conditions of wormholes are related to the acid system and the optimization of stimulation parameters in carbonate acidizing. Therefore, investigating the change in the morphology of the acid-etched wormhole after acidizing can provide theoretical conditions for on-site acidizing stimulation. To ensure the effect of acid etching wormhole, it is necessary to have oil dissolve and remove the organic scale. Therefore, the microemulsion acid can achieve the effect of acid etching wormhole and dissolving asphalt at the same time.

#### 4.2.1. Experimental Materials and Methods

**Figure 14:**
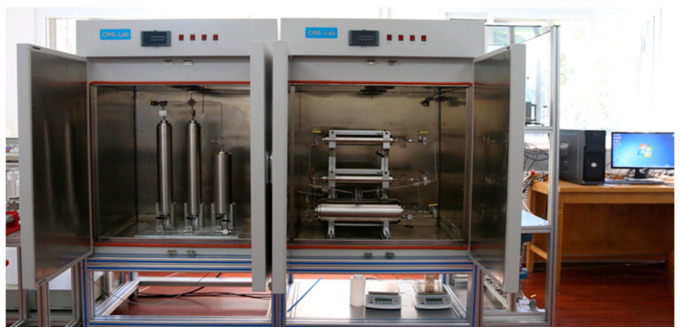
High-temperature and high-pressure driving device for acidizing.

**Figure 15:**
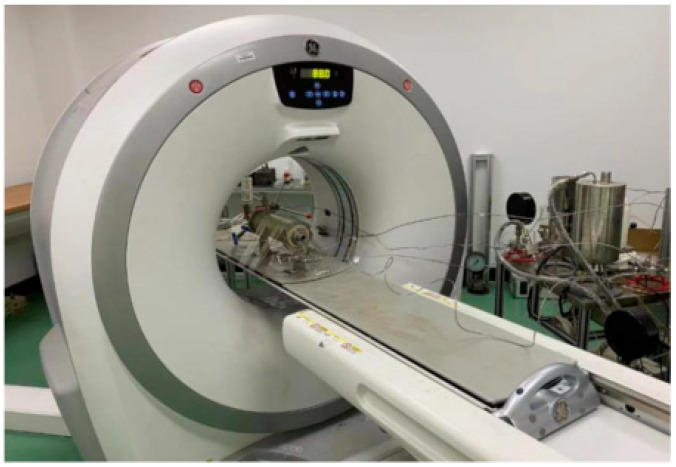
Micron precision CT scanner.

The experimental material uses 10.5% Fecl_2_ solution to damage the core, and then 15% sodium hydroxide solution is injected in reverse alternately to simulate the damage of iron scale to the core. After simulated iron scale damage, the core is put into a beaker, and the mass ratio of asphalt to heptane solution is 1:1. The specific damage process can refer to Wang‘s research [5]. The damaged core is placed into the core holder of the high-temperature and high-pressure driving device for acidizing (Figure 14). The injection rate was controlled, and the temperature was 90 °C until the breakthrough pressure drop became 0 MPa. The relevant data, such as leak-off, breakthrough volume and pressure, were recorded. After the damage was relieved, the core was washed with water, and CT was scanned to observe the wormhole morphology (Figure 15).

The experimental steps are as follows: (1) Preheat the equipment and intermediate container to 90 °C, place the core in the core holder; (2) Put the configured acid into the intermediate container and empty the pipeline air; (3) The confining pressure is slowly loaded to 2 MPa, and the confining pressure value is always greater than the core inlet pressure of 1.5~2.0 MPa during the experiment; (4) Driving the treatment fluid until the breakthrough pressure drop becomes 0, and recording the relevant data such as leak off, breakthrough volume and breakthrough pressure; (5) CT scan was performed on the core after acidizing to observe the morphology of the wormhole and analyze the acidizing effect.

#### 4.2.2. Experimental Results and Analysis

**Figure 16:**
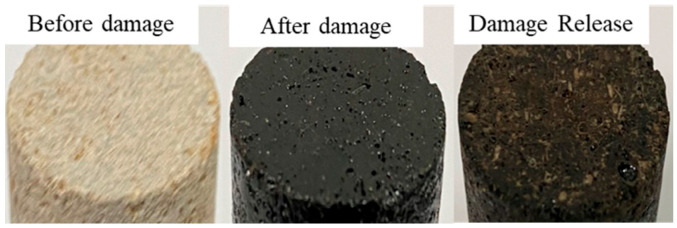
Mixed scale damage and removal.

The experimental results show that different acid treatment solutions can form acid-etched wormhole es to relieve the mixed scale damage of carbonate rock core to a certain extent. The HCl treatment solution can only dissolve the inorganic scale. It can be observed from the surface of the inlet end that the amount of asphalt adhesion after emulsified acid and microemulsion acid treatment is significantly reduced (Figure 16).

**Figure 17:**
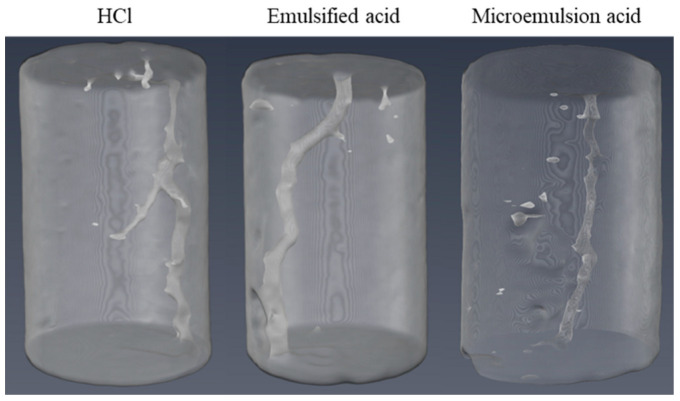
Mixed scale damage and removal.

The experimental data and results of the acid-driving experiment are shown in Table 5. PV_bt_ is the acid consumption when the wormhole breaks through the core outlet (PV_bt_ = acid consumption/pore volume). When the propagation distance of the wormhole is the same, the amount of acid used in HCl is the most. The amount of acid used when HCl breaks through the core outlet is 7.12 PV. The amount of acid used when the microemulsion acid breaks through the core outlet is the lowest at 5.14 PV. The acidizing effect of different acid fluids on carbonate rock cores after mixed scale damage is shown in Figure 17. According to the characteristics of three-dimensional wormhole graphics, there are two kinds of wormhole shapes in the cores after acidizing three groups of acid fluids: branch wormhole and main wormhole. The core treated with HCl alone shows branch wormholes and the ineffective acidizing treatment is more serious, resulting in excessive acid consumption and limited stimulation effect. Due to the inability of HCl to dissolve organic scale, the channel diameter of the main wormhole was sometimes wide and sometimes narrow. A main wormhole is formed in the core after emulsified acid treatment, and there is almost no branch wormhole, and the channel diameter of the main wormhole is larger. Due to the relatively weak dissolution ability of emulsified acid for organic scale, the morphology of the main wormhole formed is more tortuous, but the consumption is significantly reduced compared with HCl treatment. A thin and straight main wormhole was formed in the core after microemulsion acid treatment. This is because microemulsion acid has a strong dissolution ability for both organic scale and inorganic scale, so the spatial distribution of scale in the core has less hindrance to acid flow. The strong scale removal ability enables the microemulsion acid to fully dissolve organic scale, inorganic scale, and carbonate minerals. The lower acid-rock reaction rate helps the microemulsion acid form a slender main wormhole inside the core, eventually leading to the higher acidizing and plugging removal efficiency of the microemulsion acid compared with other acid systems.

## 5. Conclusions

Based on the retarding ability of emulsified acid and the stable structure of microemulsion, this study optimizes the ratio of surfactants and cosurfactants by optimizing the oil phase reagent. It prepares an acid-in-oil microemulsion acid that can dissolve organic scale and inorganic scale simultaneously and has a high solubilization ability for HCl. The acid-rock reaction kinetics, scale dissolving ability and plugging removal performance of microemulsion acid in carbonate reservoirs were systematically studied, and the following conclusions were obtained.

(1) Solvesso 150 with low toxicity, flash point of 69 °C and ability to dissolve scale of 0.0441 g/min at 90 °C was selected as the oil phase. The ratio of surfactant and the ratio of surfactant to cosurfactant were optimized according to the ability to solubilize HCl. When NP−4: OP−10: A = 3: 3: 4, the system had the strongest ability to solubilize hydrochloric acid, which solved the problem of low H^+^ concentration.
(2) The particle size of microemulsion acid is smaller than that of emulsified acid in the external oil phase, further reducing the diffusion rate. The mass transfer coefficient of microemulsion acid is 3.2 × 10^−8^ cm^2^/s at 60 °C, which is one order of magnitude smaller than that of emulsified acid. It is limited by the mass transfer process in the acid-rock reaction.
(3) Microemulsion acid shows good dissolution performance in dissolving both organic and inorganic scales, and the combined solubility of mixed scales can reach 98.28%. The stronger scale dissolving ability and lower acid rock reaction rate enable microemulsion acid to form a thin and straight main wormhole, thus enhancing the acidizing and plugging removal efficiency.

## Figures and Tables

**Table 1:** XRD results of the experimental core.

Number	Mineral Content/%				Clay Minerals and Content/%
	Calcite	Dolomite	Quartz	Clay Minerals	
1	96.8	0.0	1.4	1.8	Illite 100%
2	96.9	0.0	1.2	1.9	Illite 100%
3	97.5	0.0	1.0	1.5	Illite 100%
4	97.8	0.0	1.1	1.1	Illite 100%
5	94.5	0.0	3.0	2.5	Illite 100%
Average	96.7	0.0	1.5	1.8	Illite 100%

**Table 2:** Experimental results of inorganic scale dissolution by four liquids.

Liquid	Before Reaction/g	After Reaction/g	Dissolution/%
HCl	2.0731	0.1152	94.44
S150	2.0028	1.9945	0.41
Emulsified acid	2.0193	0.1540	92.37
Microemulsion acid	2.0249	0.1201	94.07

**Table 3:** Experimental results of organic scale dissolution by four liquids.

Liquid	Before Reaction/g	After Reaction/g	Dissolution/%
HCl	2.1074	2.1024	0.24
S150	2.0849	1.6309	21.78
Emulsified acid	2.1405	1.9881	7.12
Microemulsion acid	2.1054	1.6839	20.02

**Table 4:** Experimental results of maxed scale dissolution by four liquids.

Liquid	Before Reaction/g	After Reaction/g	Dissolution/%
HCl	2.0894	2.0324	2.73
S150	2.1142	1.0632	49.69
Emulsified acid	2.0149	1.6893	16.16
Microemulsion acid	2.0926	0.0360	98.28

**Table 5:** Experimental results of maxed scale dissolution by four liquids.

Number	Treating Fluid	Injection Rate/mL/min	PV_bt_
1	HCl	2	7.12
2	Emulsified acid	2	6.11
3	Microemulsion acid	2	5.14

## Data Availability

Not applicable.

## Sample Availability

Samples of the compounds are available from the authors.
